# Suppression of A-to-I RNA-editing enzyme ADAR1 sensitizes hepatocellular carcinoma cells to oxidative stress through regulating Keap1/Nrf2 pathway

**DOI:** 10.1186/s40164-024-00494-7

**Published:** 2024-03-11

**Authors:** Houhong Wang, Xiaoyu Wei, Lu Liu, Junfeng Zhang, Heng Li

**Affiliations:** 1 Department of General Surgery, The First Hospital Affiliated to Fuyang Normal University, Fuyang, 236006 Anhui China; 2 Department of Infectious Diseases, Yongchuan Hospital of Chongqing Medical University, Chongqing, 402160 China; 3 Department of Endocrinology, The Affiliated Nantong Hospital of Shanghai Jiao Tong University, Nantong, 226001 Jiangsu China; 4 Department of Radiology, General Hospital of Western Theater Command of PLA, Chengdu, 610083 Sichuan China; 5 Department of Comprehensive Surgery, Anhui Provincial Cancer Hospital, West District of The First Affiliated Hospital of USTC, Hefei, 230031 Anhui China; 6 Department of General Surgery, The Affiliated Bozhou Hospital of Anhui Medical University, Bozhou, 236800 Anhui China

**Keywords:** ADAR1, Hepatocellular carcinoma, Oxidative stress, Survival, Keap1/Nrf2, Reactive oxygen species

## Abstract

**Background:**

A-to-I RNA editing is an abundant post-transcriptional modification event in hepatocellular carcinoma (HCC). Evidence suggests that adenosine deaminases acting on RNA 1 (ADAR1) correlates to oxidative stress that is a crucial factor of HCC pathogenesis. The present study investigated the effect of ADAR1 on survival and oxidative stress of HCC, and underlying mechanisms.

**Methods:**

ADAR1 expression was measured in fifty HCC and normal tissues via real-time quantitative PCR, and immunohistochemistry. For stable knockdown or overexpression of ADAR1, adeno-associated virus vectors carrying sh-ADAR1 or ADAR1 overexpression were transfected into HepG2 and SMMC-7721 cells. Transfected cells were exposed to oxidative stress agonist tBHP or sorafenib Bay 43-9006. Cell proliferation, apoptosis, and oxidative stress were measured, and tumor xenograft experiment was implemented.

**Results:**

ADAR1 was up-regulated in HCC and correlated to unfavorable clinical outcomes. ADAR1 deficiency attenuated proliferation of HCC cells and tumor growth and enhanced apoptosis. Moreover, its loss facilitated intracellular ROS accumulation, and elevated Keap1 and lowered Nrf2 expression. Intracellular GSH content and SOD activity were decreased and MDA content was increased in the absence of ADAR1. The opposite results were observed when ADAR1 was overexpressed. The effects of tBHP and Bay 43–9006 on survival, apoptosis, intracellular ROS accumulation, and Keap1/Nrf2 pathway were further exacerbated by simultaneous inhibition of ADAR1.

**Conclusions:**

The current study unveils that ADAR1 is required for survival and oxidative stress of HCC cells, and targeting ADAR1 may sensitize HCC cells to oxidative stress via modulating Keap1/Nrf2 pathway.

## Background

Hepatocellular carcinoma (HCC) that occupies nearly 90% of primary liver cancer is one of the most lethal and prevalent human cancers globally [1]. Most HCCs occur in the context of cirrhosis, and the most frequent etiology is nonalcoholic fatty liver disease, alcohol-related liver disease, and HBV/HCV infection [2]. Because of delayed diagnosis and limited efficacy of existing treatment options, prognosis is still undesirable, with a five-year survival rate of only 15% [3]. Targeted therapy especially tyrosine kinase inhibitor sorafenib has notably improved the systemic treatment of advanced HCC [4]. Since sorafenib was approved as the first-line standard of care, other targeted drugs exhibited clinical benefits in phase III trials, including the first-line lenvatinib and the second-line regorafenib, cabozantinib and ramucirumab, etc. [5–8]. Nevertheless, all of these agents only modestly prolong median survival (2–3 months) and confer a low response rate. Understanding the mechanisms that control HCC resistance to targeted drugs is critical for guiding the efforts to sensitize HCC cells to above promising drugs [9–12].

HCC arises from chronic tissue damage correlated to oxidative stress that is triggered by reactive oxygen species (ROS) production [13]. Enhanced intrinsic or adaptive antioxidant ability assists tumor cells survive oxidative injury [14]. Nrf2 is a major antioxidant transcription factor, which transcriptionally mediates the antioxidant enzyme gene repertoire, thereby controlling critical biological processes associated with ROS decline and defense against oxidative stress [15]. Targeted regulation of Nrf2 can be applied for treating human chronic diseases, especially cancers [16]. Under a normal condition, Nrf2 is constitutively low expressed in the cytoplasm because it is ubiquitinated and degraded by Keap1-triggered proteasome [17]. The disruption of Keap1 results in enhanced cellular Nrf2 activity [18]. Under an oxidative stress condition, cytoplasmic Nrf2 is translocated to the nucleus, thus binding to antioxidant response elements (AREs) present in cytoprotective target genes and promoting their transcription [19]. Nrf2 frequently exhibits up-regulation and activation in HCC, which correlates to malignant phenotypes and undesirable clinical outcomes [20]. Adenosine deaminase acting on RNA (ADAR) catalyzes the transformation of adenosine (A) to inosine (I) in double-stranded RNA (dsRNA) substrates, which is an important process with arresting physiological significance [21]. HCC exhibits a severely disrupted balance of A to I RNA editing [22]. Three ADAR enzymes (ADAR1-3) exist in humans. ADAR1 and ADAR2 catalyze all common A-to-I editing events; oppositely, ADAR3 had no recorded deaminase activity [23]. ADAR1 is overexpressed in human cancers; in contrast, ADAR2 is down-regulated in e.g. glioblastoma, and thus results in malignant phenotypes [24]. Accumulated evidence has demonstrated the crucial function of ADAR1 in cancer progression and therapy, such as anti-tumor immunity [25], metastasis [26], and stemness [27]. In HCC, ADAR1 manipulates the A to I imbalance through abnormal expression [22]. ADAR1 up-regulation correlates to an increased risk of liver cirrhosis, postoperative relapse and undesirable prognosis [28]. ADAR1 deficiency triggers NFκB and interferon signaling dependent liver inflammatory response and fibrosis [29]. Additionally, ADAR1 heightens adhesion of HCC cells to extracellular matrix through ITGA2 up-regulation [30]. Several studies have demonstrated the correlation between ADAR1 and oxidative stress. Takizawa et al. reported that reduction of ADAR1 expression exposed to cigarette smoke heightens susceptibility to oxidative stress [31]. Siew et al. found that ADAR1(p150) is localized to cytoplasmic stress granules in HeLa cells after oxidative or interferon-induced stress [32]. In the Wang et al.’s study, ADAR1 mRNA and protein expression are both increased following H_2_O_2_-induced oxidative stress in neonatal cardiac myocytes [33]. Despite this, the role of ADAR1 in oxidative stress of HCC cells remains unclear. The current study hypothesized that ADAR1 loss sensitized HCC cells to oxidative stress via mediating Keap1/Nrf2 pathway.

## Materials and methods

### Patient specimens

Fifty HCC tissues and matched adjacent normal tissues were harvested from deidentified HCC cases who underwent hepatectomy at The Anhui Provincial Cancer Hospital. The protocols implemented in our study were approved by Anhui Provincial Cancer Hospital (2023-35), and each patient provided written informed consent.

### Real-time quantitative PCR

Total RNA was extracted with RNA easy mini kit (Invitrogen, USA), and cDNA preparation was implemented utilizing PrimeScript RT Master Mix (Takara, China). Real-time PCR was carried out utilizing ChamQ SYBR qPCR Master Mix (Vazyme, China) following the manufacturer’s instructions. The primer sequences included: ADAR1, 5′-CTGAGACCAAAAGAAACGCAGA-3′ (forward), and 5′-GCCATTGTAATGAACAGGTGGTT-3′ (reverse); GAPDH, 5′-TATGATGATATCAAGAGGGTAGT-3′ (forward), and 5′-ATGGAAGACGGGAGATTCACAT-3′ (reverse). The relative expression was computed with 2^−ΔΔCt^ approach and normalized to endogenous GAPDH.

### Immunohistochemistry

Tissues were fixed in formalin and embedded in paraffin. Four-μm-thick sections were mounted on poly-l-lysine-coated slides. Afterwards, the slides were dewaxed in xylene and rehydrated with a gradient of ethanol and distilled water. To quench endogenous peroxidase activity, the sections were exposed to 3% hydrogen peroxide for 10 min at room temperature, followed by antigen retrieval. Thereafter, incubation with primary antibodies against ADAR1 (1/100; ab168809; Abcam, USA), Ki-67 (1/100; ab21700; Abcam), Caspase-3 (1/100; ab32499; Abcam), Keap1 (1/200; ab227828; Abcam), and Nrf2 (1/100; ab137550; Abcam) was conducted at 4 °C overnight. Then, the sections were probed with HRP anti-rabbit IgG antibody (1/200; ab288151; Abcam), stained with DAB, and nucleated with hematoxylin, followed by dehydration with a gradient of ethanol and sealing with neutral gum. Protein expression was quantified with ImageJ software.

### Cell culture

Hepatocytes (L-02) as well as HCC cells (Huh7, HepG2, Hep3B, and SMMC-7721) were purchased from the Chinese Academy of Sciences (China) or ATCC (USA). HepG2 and Hep3B cells were cultivated in Minimum Essential Medium (HyClone, USA); L-02 cells were grown in Dulbecco’s modified Eagle’s medium (HyClone); and SMMC-7721 cells were cultured in Roswell Park Memorial Institute-1640 medium. Above media were supplemented with 10% fetal bovine serum (FBS) (HyClone), and 1% penicillin/streptomycin (HyClone). All the cells were grown in an incubator of 5% CO_2_ at 37 °C.

### Immunoblot analysis

Total protein extracts were isolated using RIPA lysis buffer (Sigma, USA), and were quantified with bicinchoninic acid (BCA) kit (Pierce, USA). Afterwards, extracted proteins were separated via SDS/PAGE, and transferred onto PVDF membranes. The membranes were then blocked by TBST supplemented with 5% skimmed milk. Antibodies listed below were utilized for incubating the membranes, comprising ADAR1 (1/1000; ab168809; Abcam), β-actin (1/5000; ab179467; Abcam), Keap1 (1/2000; ab227828; Abcam), and Nrf2 (1/500; ab137550; Abcam), and HRP anti-rabbit IgG antibody (1/2000; ab288151; Abcam).

### Establishment of stable cell lines and drug administration

To stably silence ADAR1, adeno-associated virus vector that carried small hairpin RNA (shRNA) targeting human ADAR1 (sh-ADAR1) and human nonsense control shRNA (sh-NC) (Shanghai GenePharma Co. Ltd. (China)) were utilized to transfect cells following the manufacturer’s instructions. In addition, ADAR1 was overexpressed through transfection of adeno-associated virus vector of ADAR1 overexpression (OE-ADAR1) (GenePharma). At 48 h post transfection, transfection effect was verified. Then, transfected cells were exposed to 100 μmol/L Tert-butyl Hydroperoxide (tBHP; Sigma-Aldrich, USA) or 2.5 μmol/L sorafenib Bay 43–9006 (Sigma-Aldrich), as previously described [34].

### Cell viability assay

Cells were seeded into a 96-well plate (2 × 10^3^ cells/well), and cultivated for indicated times. 10 μL cell counting Kit-8 (CCK-8) (MedchemExpress, USA) was added into each well. Thereafter, the cells continued to cultivate for 2 h. The absorbance at 450 nm was read with Microplate Reader (Thermo Scientific, USA).

### 5-ethynyl-2′-deoxyuridine (EdU) staining

Cells were seeded into a 24-well plate (2 × 10^4^ cells/well). The original culture medium was discarded, and 300 μL medium supplemented with 50 μM EdU (RiboBio China) was added into each well. After cultivating for 2 h, the EdU medium was discarded, and the cells were rinsed with PBS. Thereafter, the cells were fixed with 4% paraformaldehyde (Sigma-Aldrich) for 30 min at room temperature, followed by quench with 2 mg/mL glycine solution, permeabilization with 0.5% Triton X-100 for 10 min. Then, they were dyed with Apollo dye reagent for 30 min. EdU-positive cells were captured with an Olympus FSX100 microscope (Olympus, Japan).

### Tumor xenograft

BALB/c nude mice (5-week-old, 16–18 g; Beijing Vital River Laboratory Animal Technology Co., Ltd., China) were fed with a 12-h light/dark cycle. The mice were randomly separated into sh-NC and sh-ADAR1 groups (n = 6 each group). 1 × 10^5^ luciferase-tagged SMMC-7721 cells stably transfected with sh-NC or sh-ADAR1 were inoculated into the armpit of mice. At 28 days after inoculation, bioluminescence signals were detected. The mice were sacrificed, and the tumors were excised and gathered. Tumor volume was calculated according to the equation (L × W^2^)/2. The animal experiments were approved by the Animal Ethics Committee of Anhui Provincial Cancer Hospital (2023-35).

### Flow cytometry

Apoptosis was assayed with Annexin V-FITC apoptosis detection kit (Thermo Scientific, USA). In brief, cells (1 × 10^6^ cells/mL) were resuspended in 1 × binding buffer. Afterwards, 2 × 10^5^ cells were exposed to 10 μL Annexin V-FITC as well as 10 μL 7-aminoactinomycin D for 15 min away from light. Samples were tested on a FACSCanto II flow cytometer (BD, USA).

### RNA sequencing (RNA-seq) and data analysis

Total RNA was extracted by use of TRIzol reagent kit (Invitrogen, USA) following the manufacturer’s specification. RNA quality was evaluated based on an Agilent 2100 Bioanalyzer (Agilent Technologies, USA), with subsequent validation via RNase-free agarose gel electrophoresis. Eukaryotic mRNA was enriched by oligo(dT) beads, while prokaryotic mRNA was enriched by removal of rRNA. Afterwards, the enriched mRNA was segmented into short fragments utilizing fragmentation buffer and reverse-transcribed into cDNA utilizing random primers. The second-strand cDNA was synthesized, and the cDNA fragment was purified utilizing a QiaQuick PCR extraction kit (Qiagen, The Netherlands), with subsequent end repair, A base treatment, and ligation to Illumina sequencing adapters. The size of the ligation products was chosen via agarose gel electrophoresis, amplified by PCR, and sequenced utilizing Illumina NovaSeq6000. Differentially expressed genes between groups were screened under the criteria of |log2 fold change|> 0.585 and adjusted p < 0.05, and functional enrichment analysis was carried out via clusterProfiler package.

### Intracellular ROS detection

Intracellular ROS level was measured with DCFH-DA (Beyotime, China). Briefly, cells (5 × 10^4^ cells/mL) were seeded into a 12-well plate, and exposed to 10 μM DCFH-DA for 30 min at 37 °C. Afterwards, they were washed with PBS, and resuspended in ice-cold PBS away from light. The intracellular ROS was photographed under a fluorescence microscope.

### Co-immunoprecipitation (Co-IP)

Cells were lysed on ice for 4 h, and cell lysate was centrifuged at 13,000 rpm for 10 min. The supernatant was harvested and pre-incubated with immunopure immobilized protein A for removing non-specific proteins binding to the beads. Then, the supernatant was incubated with IgG or ADAR1 antibody (1/1000; ab168809; Abcam) for 1 h, followed by protein A agarose beads with gentle agitation at 4 °C overnight. Immunoblot analysis was conducted by ADAR1 (1/1000; ab168809; Abcam), β-actin (1/5000; ab179467; Abcam), Keap1 (1/2000; ab227828; Abcam) and Nrf2 (1/500; ab137550; Abcam) antibodies.

### Immunofluorescence

Cells were plated on glass coverslips in a 24-well plate (1 × 10^4^ cells/well). After fixing with 4% paraformaldehyde for 15 min, the cells were permeabilized utilizing 0.25% TritonX-100 for 3 min, with subsequent blockade in 1% BSA at room temperature for half one hour. The coverslips were incubated with primary antibodies against ADAR1 (1/100; ab168809; Abcam), Keap1 (1/200; ab139729; Abcam), and Nrf2 (1/100; ab137550; Abcam) overnight at 4 °C, with subsequent incubation with goat anti-rabbit IgG H&L Alexa Fluor® 488 (1/200; ab150077; Abcam) or Alexa Fluor® 647 (1/200; ab150083; Abcam) at room temperature for 1 h. The nuclei were stained by DAPI. Images were obtained utilizing a fluorescence microscope.

### Detection of glutathione (GSH), and malondialdehyde (MDA) content and superoxide dismutase (SOD) activity

According to the manufacturer’s instructions, intracellular content of GSH and MDA and activity of SOD were detected though GSH, MDA and SOD kits, respectively (Abbkine, China). Through BCA approach, protein concentration was assessed in cell lysates to normalize GSH and MDA content as well as SOD activity. GSH and MDA content was separately measured at 420 nm and 532 nm with spectrometer, while SOD activity was tested at 550 nm.

### Transmission electron microscopy (TEM)

Cells were digested with 0.25% trypsin and immobilized in 2.5% glutaraldehyde solution for 12 h. Subsequently, they were postfixed in 1% aqueous osmium tetroxide, dehydrated in a series of graded ethanol with propylene oxide, embedded in epoxy resin, with incubation at 60 °C for 48 h. After staining with toluidine blue, ultrathin sections were stained with 2% uranyl acetate and lead citrate. Ultrastructural images were acquired utilizing a transmission electron microscope (Tokyo, Japan).

### Statistical analysis

The TIMER2.0 (http://timer.cistrome.org/) tool was employed to assess ADAR1 expression in tumors and matched normal tissues across pan-cancer from The Cancer Genome Atlas (TCGA) [35]. Additionally, associations between ADAR1 expression and overall survival across pan-cancer were evaluated via univariate cox regression analysis. Student’s t-test was employed for determining the differences between two groups, with one-way analysis of variance (ANOVA) for the differences between ≥ 3 groups. P ≤ 0.05 was considered statistical significance.

## Results

### ADAR1 exhibits high expression in HCC and correlates to unfavorable clinical outcome

**Fig. 1:**
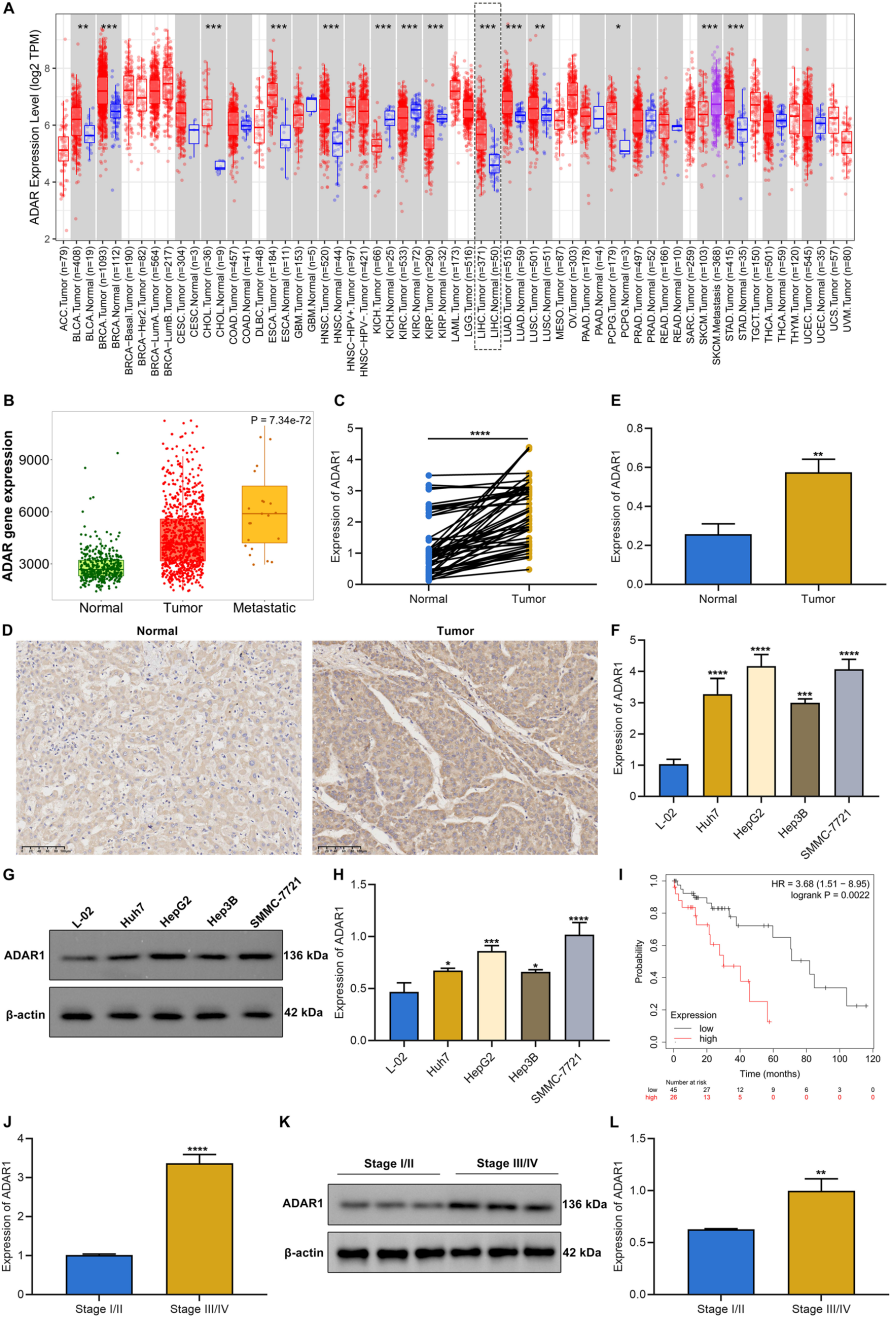
ADAR1 exhibits high expression in HCC and correlates to clinical outcome. **A** Pan-cancer analysis of ADAR1 expression in tumors and matched normal tissues from TCGA database via TIMER2.0 tool. **B** Distribution of ADAR1 expression across normal, tumor and metastatic tissues from TCGA database. **C** Paired comparison of ADAR1 expression in HCC tumors and matched normal tissues. **D**, **E** Immunohistochemistry of ADAR1 expression in tumors and matched normal tissues. **F** Comparison of ADAR1 expression detected by real-time quantitative PCR in hepatocytes (L-02) with HCC cells (Huh7, HepG2, Hep3B, and SMMC-7721). **G**, **H** Immunoblot analysis of ADAR1 expression in hepatocytes and HCC cells. **I** Survival curves of patients with high and low ADAR1 expression in HCC patients. **J**–**L** Real-time quantitative PCR and immunoblot analysis of ADAR1 expression in stage I/II and stage III/IV patients. *p < 0.05; **p < 0.01; ***p < 0.001; ****p < 0.0001

First, comparison of ADAR1 expression in tumors with matched normal tissues from TCGA database was carried out across pan-cancer. Intriguingly, ADAR1 exhibited remarkably high expression in most cancer types, comprising bladder cancer (BLCA), breast cancer (BRCA), cholangiocarcinoma (CHOL), esophageal carcinoma (ESCA), head and neck squamous cell carcinoma (HNSC), liver hepatocellular carcinoma (LIHC), lung adenocarcinoma (LUAD), lung squamous cell carcinoma (LUSC), pheochromocytoma and paraganglioma (PCPG), and stomach adenocarcinoma (STAD) (Fig. 1A). Oppositely, ADAR1 displayed reduced expression in kidney cancer (kidney chromophobe (KICH), kidney renal clear cell carcinoma (KIRC), and kidney renal papillary cell carcinoma (KIRP)). The present study focused on the roles of ADAR1 in HCC. ADAR1 expression was higher in metastatic HCC in comparison to primary tumors (Fig. 1B). Paired comparison of ADAR1 expression was then implemented in fifty HCC tumors and matched normal tissues. As expected, elevated expression of ADAR1 was found in tumors than normal specimens (Fig. 1C–E). Additionally, ADAR1 expression displayed up-regulation in HCC cells (Huh7, HepG2, Hep3B, and SMMC-7721) compared with hepatocytes (L-02) (Fig. 1F–H). Among HCC cells, SMMC-7721 and HepG2 cells had the highest expression of ADAR1, which were employed for subsequent analysis. Survival curves showed that patients with ADAR1 up-regulation presented poorer OS relative to those with its down-regulation (Fig. 1I). Thus, ADAR1 might act as a risk factor of HCC prognosis. In addition, we compared the expression of ADAR1 between stage I/II and stage III/IV patients. The results showed that higher ADAR1 expression was found in stage III/IV relative to stage I/II (Fig. 1J–L). Altogether, ADAR1 expression exhibited up-regulation in HCC and was correlated to patient prognosis.

### ADAR1 loss attenuates proliferative capacity of HCC cells

**Fig. 2:**
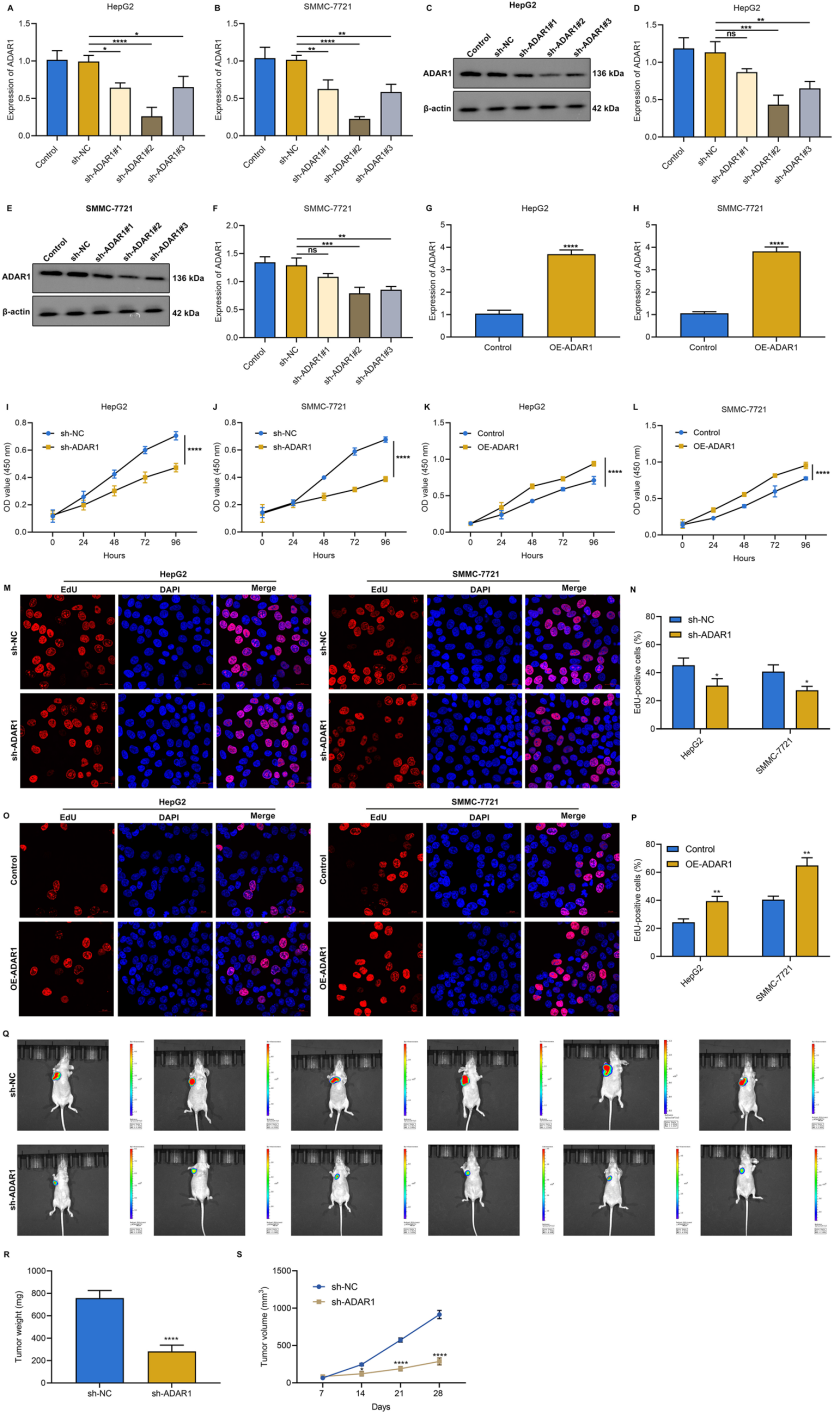
ADAR1 loss attenuates proliferative capacity of HCC cells. **A**, **B** Real-time quantitative PCR of ADAR1 expression in HepG2 and SMMC-7721 cells transfected with sh-NC or three shRNAs against ADAR1. **C**–**F** Immunoblot analysis of ADAR1 expression in HepG2 and SMMC-7721 cells transfected with sh-NC or three shRNAs against ADAR1. **G**, **H** Real-time quantitative PCR of ADAR1 expression in HepG2 and SMMC-7721 cells with ADAR1 overexpression transfection. **I**–**L** CCK-8 for cell viability of HepG2 and SMMC-7721 cells with sh-ADAR1 or ADAR1 overexpression transfection. **M**–**P** EdU staining for proliferation of HepG2 and SMMC-7721 cells with sh-ADAR1 or ADAR1 overexpression transfection. Bar, 20 μm. **Q** Representative images of BALB/c nude mice at day 28 after inoculating sh-NC or sh-ADAR1 luciferase-tagged SMMC-7721 cells. **R**, **S** Tumor weight and tumor growth curve. Ns: no significance; *p < 0.05; **p < 0.01; ***p < 0.001; ****p < 0.0001

For stable knockdown of ADAR1, three shRNAs targeting human ADAR1 were transfected into HepG2 and SMMC-7721 cells. Real-time quantitative PCR (with GAPDH as the internal control) and immunoblot analysis confirmed that sh-ADAR1#2 exhibited the best silencing efficacy among three specific shRNAs, which was employed for subsequent analysis (Fig. 2A–F). Additionally, ADAR1 was remarkably overexpressed by adeno-associated virus vector in HepG2 and SMMC-7721 cells (Fig. 2G, H). CCK-8 assay demonstrated that proliferative phenotype of HepG2 and SMMC-7721 cells was mitigated by ADAR1 loss (Fig. 2I, J). The opposite results were found when ADAR1 was overexpressed (Fig. 2K, L). Additionally, in the absence of ADAR1, the percentages of EdU-positive HepG2 and SMMC-7721 cells were attenuated (Fig. 2M, N). In contrast, ADAR1 overexpression increased the percentages of EdU-positive HCC cells (Fig. 2O, P). To further investigate the influence of ADAR1 knockdown on tumor growth, tumor xenograft experiment was conducted. Sh-NC or sh-ADAR1 luciferase-tagged SMMC-7721 cells were inoculated into BALB/c nude mice. After 28 days, our results demonstrated that ADAR1 loss notably mitigated the tumor volume and weight (Fig. 2Q–S). Thus, ADAR1 deficiency attenuated proliferative capacity of HCC cells.

### ADAR1 loss induces apoptosis and mediates oxidative stress in HCC cells

**Fig. 3:**
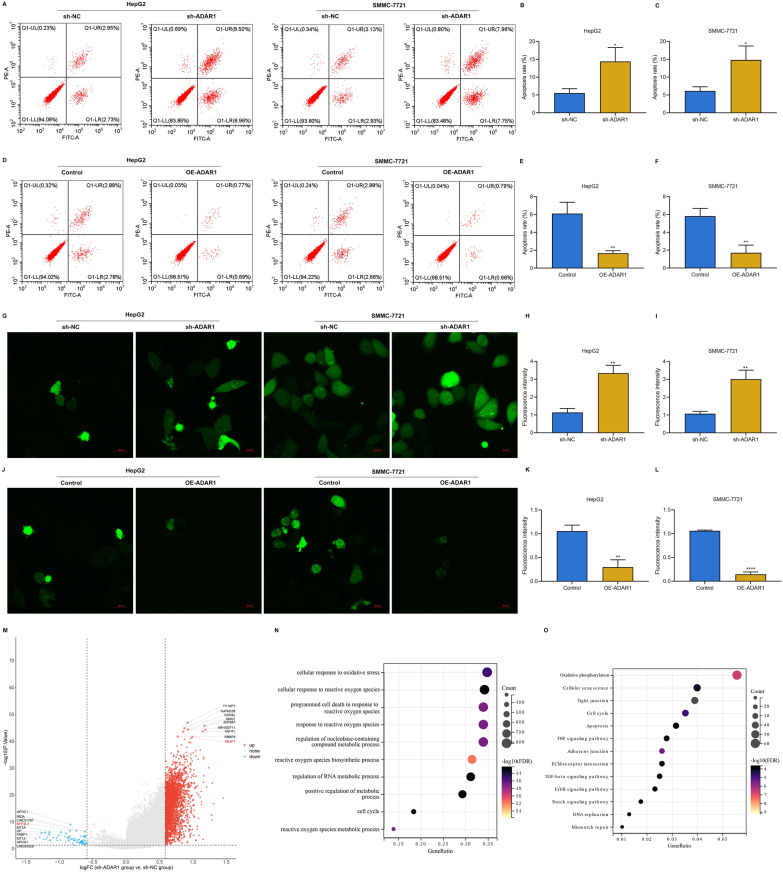
ADAR1 is responsible for apoptosis, and oxidative stress in HCC cells. **A**–**F** Flow cytometry analysis of apoptotic level of HepG2 and SMMC-7721 cells with sh-ADAR1 or ADAR1 overexpression transfection. **G**–**L** DCFH-DA fluorescent probe for measuring intracellular ROS level of HepG2 and SMMC-7721 cells with sh-ADAR1 or ADAR1 overexpression transfection. Bar, 20 μm. **M** Volcano plots of differentially expressed genes between sh-NC and sh-ADAR1 HCC cells. **N**, **O** Biological processes and KEGG pathways enriched by the differentially expressed genes. *p < 0.05; **p < 0.01; ****p < 0.0001

Then, we observed that ADAR1 deficiency facilitated apoptosis of HepG2 and SMMC-7721 cells (Fig. 3A–C). Oppositely, apoptosis was mitigated by ADAR1 overexpression (Fig. 3D–F). Through DCFH-DA fluorescent probe, intracellular ROS level was measured. Data showed that intracellular ROS level was heightened by ADAR1 deficiency (Fig. 3G–I), and was weakened by its overexpression (Fig. 3J–L). Altogether, ADAR1 loss enabled to induce apoptosis and intracellular ROS production in HCC cells. To further identify the downstream targets of ADAR1 in HCC, we carried out RNA-seq analysis on sh-ADAR1 and sh-NC HCC cells. The results demonstrated that 69 genes were down-regulated, and 2614 genes were up-regulated after ADAR1 loss (|log2 fold change|> 0.585 and adjusted p < 0.05) (Fig. 3M). Further functional enrichment analysis revealed that the differentially expressed genes were mainly associated with oxidative stress-related cellular processes and pathways (Fig. 3N, O). Therefore, we inferred that ADAR1 potentially regulated oxidative stress in HCC cells.

### ADAR1 activates Keap1/Nrf2 pathway in HCC cells

**Fig. 4:**
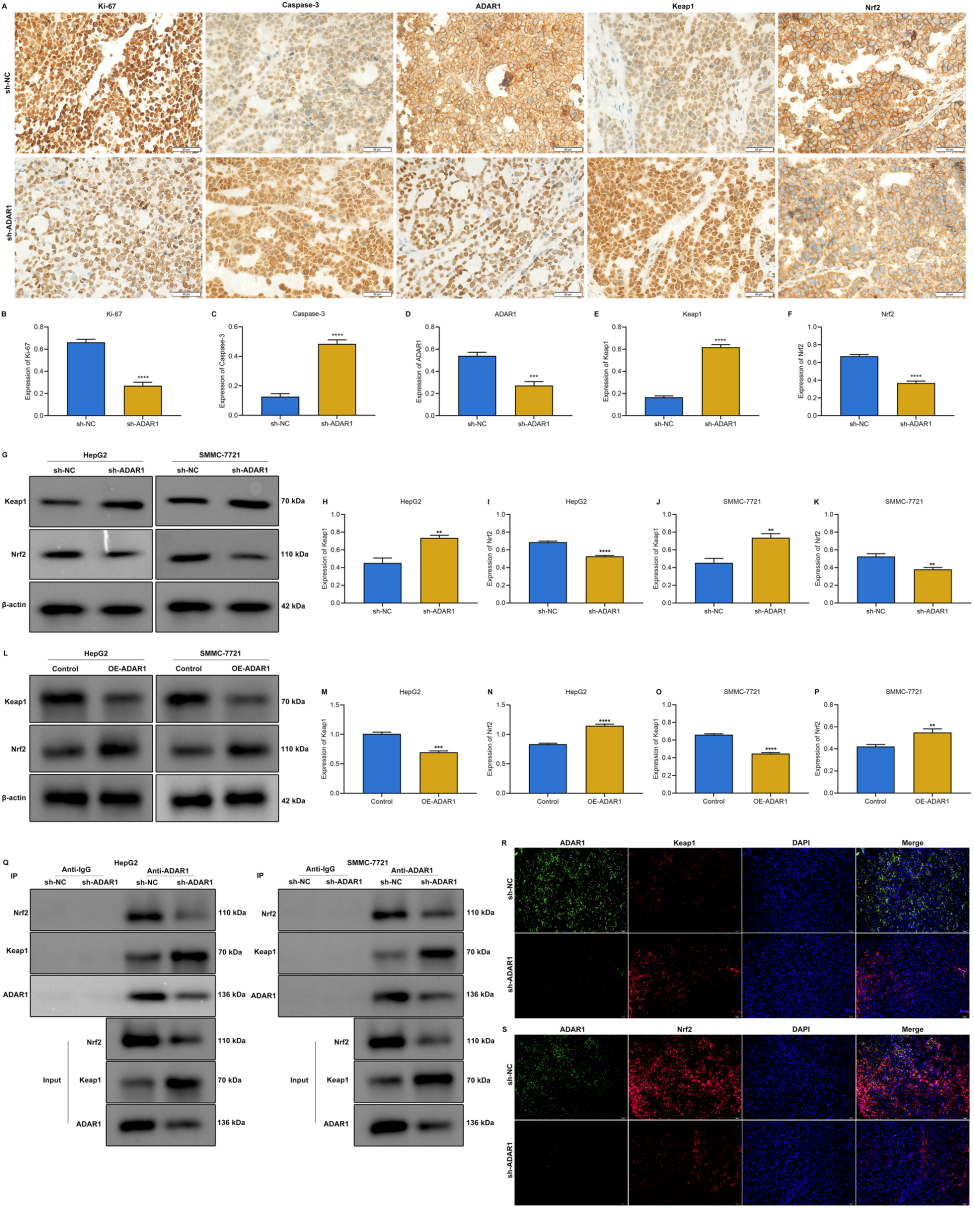
ADAR1 mediates Keap1/Nrf2 pathway in HCC. **A**–**F** Immunohistochemistry of Ki-67, Caspase-3, ADAR1, Keap1, and Nrf2 expression in mouse tumors with sh-NC or sh-ADAR1. Bar, 50 μm. **G**–**P** Immunoblot analysis of Keap1 or Nrf2 expression in HepG2 and SMMC-7721 cells with sh-ADAR1 or ADAR1 overexpression transfection. **Q** Co-IP assay of Keap1, and Nrf2 in ADAR1 precipitates in HepG2 and SMMC-7721 cells with sh-NC or sh-ADAR1. **R** Multiple immunofluorescence images of ADAR1 and Keap1 in tumor tissues from sh-NC or sh-ADAR1 mice. Bar, 10 μm. **S** Multiple immunofluorescence images of ADAR1 and Nrf2 in tumor tissues from sh-NC or sh-ADAR1 mice. Bar, 10 μm. **p < 0.01; ***p < 0.001; ****p < 0.0001

Among the differentially expressed genes, we found that Nrf2 was remarkably down-regulated, while Keap1 was remarkably up-regulated after ADAR1 loss (Fig. 3M). Keap1/Nrf2 signaling controls cellular defense against oxidative stress, thereby mediating cell survival of HCC cells [36]. Under oxidative stress, Nrf2 is released from Keap1, thereby translocating into the nucleus. Based on the evidence, we inferred that ADAR1 can regulate Keap1/Nrf2 pathway in HCC. As expected, in mouse tumors with ADAR1 knockdown, the expression of Ki-67, ADAR1, and Nrf2 was notably decreased, while the expression of Caspase-3, and Keap1 was notably elevated (Fig. 4A–F). Meanwhile, both in HepG2 and SMMC-7721 cells, ADAR1 deficiency remarkably improved Keap1 expression as well as lowered Nrf2 expression (Fig. 4G–K). The opposite findings were investigated when ADAR1 was overexpressed (Fig. 4L–P). To further validate the interactions between ADAR1 and Keap1/Nrf2, we carried out Co-IP assay. Both in HepG2 and SMMC-7721 cells, sh-ADAR1 transfection enhanced the enrichment of Keap1 as well as attenuated the enrichment of Nrf2 in ADAR1 precipitates (Fig. 4Q). In addition, in the absence of ADAR1, Keap1 expression was increased, and Nrf2 expression was attenuated in mouse tumor tissues (Fig. 4R, S). These findings confirmed that ADAR1 participated in regulating Keap1/Nrf2 signaling in HCC.

### ADAR1 deficiency enhances the inhibiting effect of oxidative stress agonist tBHP on HCC cell proliferation and its auxo-action on apoptosis

**Fig. 5:**
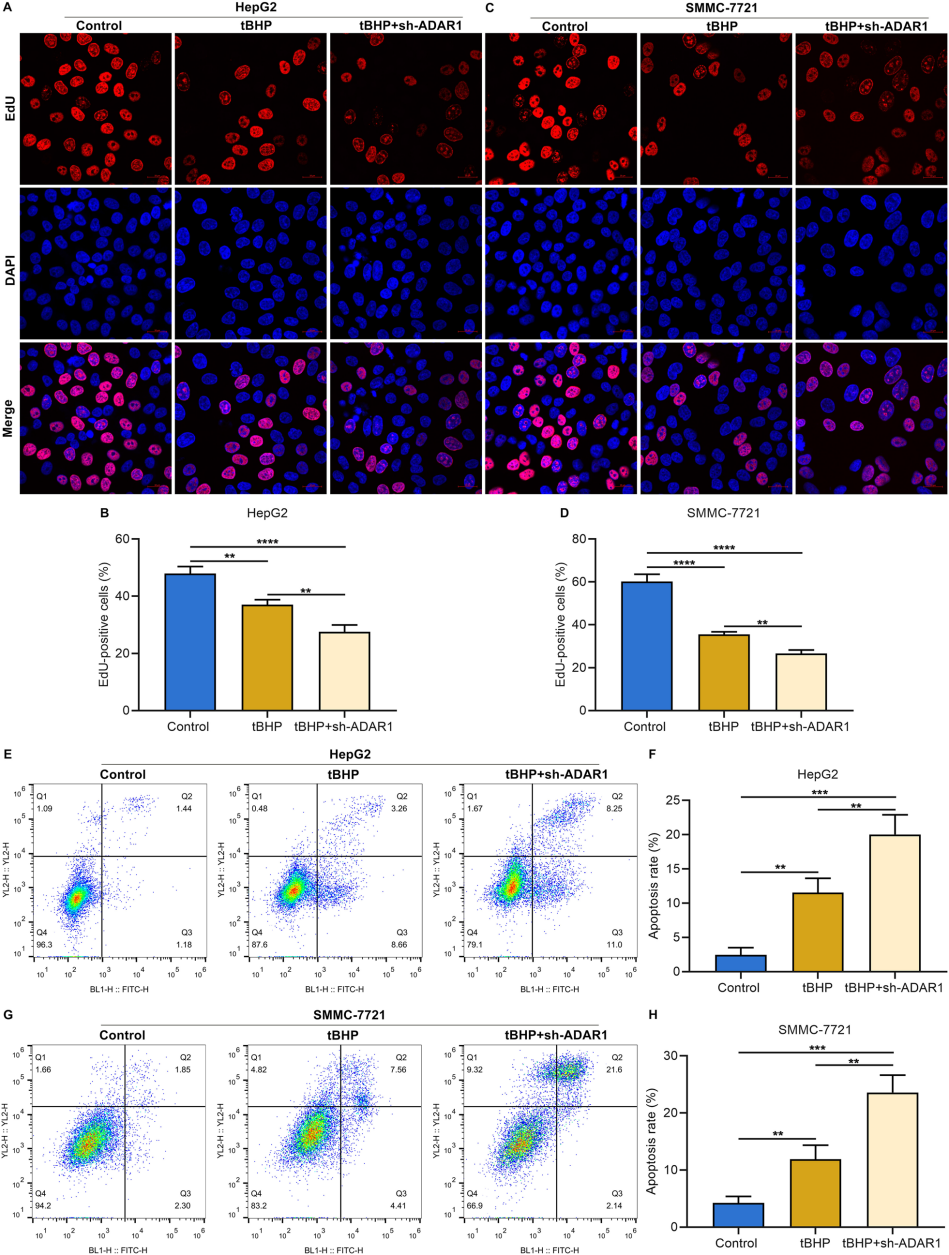
ADAR1 deficiency enhances the inhibiting effect of oxidative stress agonist tBHP on HCC cell proliferation and the promoting effect on apoptosis. **A**–**D** EdU staining for proliferation of HepG2 and SMMC-7721 cells with tBHP administration and/or sh-ADAR1 transfection. Bar, 20 μm. **E**–**H** Flow cytometry analysis of apoptotic level of HepG2 and SMMC-7721 cells with tBHP administration and/or sh-ADAR1 transfection. **p < 0.01; ***p < 0.001; ****p < 0.0001

Evidence suggests that tBHP induces oxidative stress, and thus mitigates cell viability of HCC cells [34]. As expected, administration of tBHP markedly attenuated proliferative capacity of HepG2 and SMMC-7721 cells (Fig. 5A–D). ADAR1 deficiency and tBHP treatment simultaneously mitigated HCC cell proliferation. As demonstrated by flow cytometry analysis, apoptotic level of HepG2 and SMMC-7721 cells was triggered by tBHP exposure, which was further strengthened by simultaneous suppression of ADAR1 (Fig. 5E–H). Hence, ADAR1 deficiency was capable of heightening the inhibiting effect of tBHP on HCC cell proliferation as well as its auxo-action on apoptosis.

### ADAR1 deficiency and tBHP simultaneously enhance intracellular ROS accumulation and regulate Keap1/Nrf2 pathway in HCC cells

**Fig. 6:**
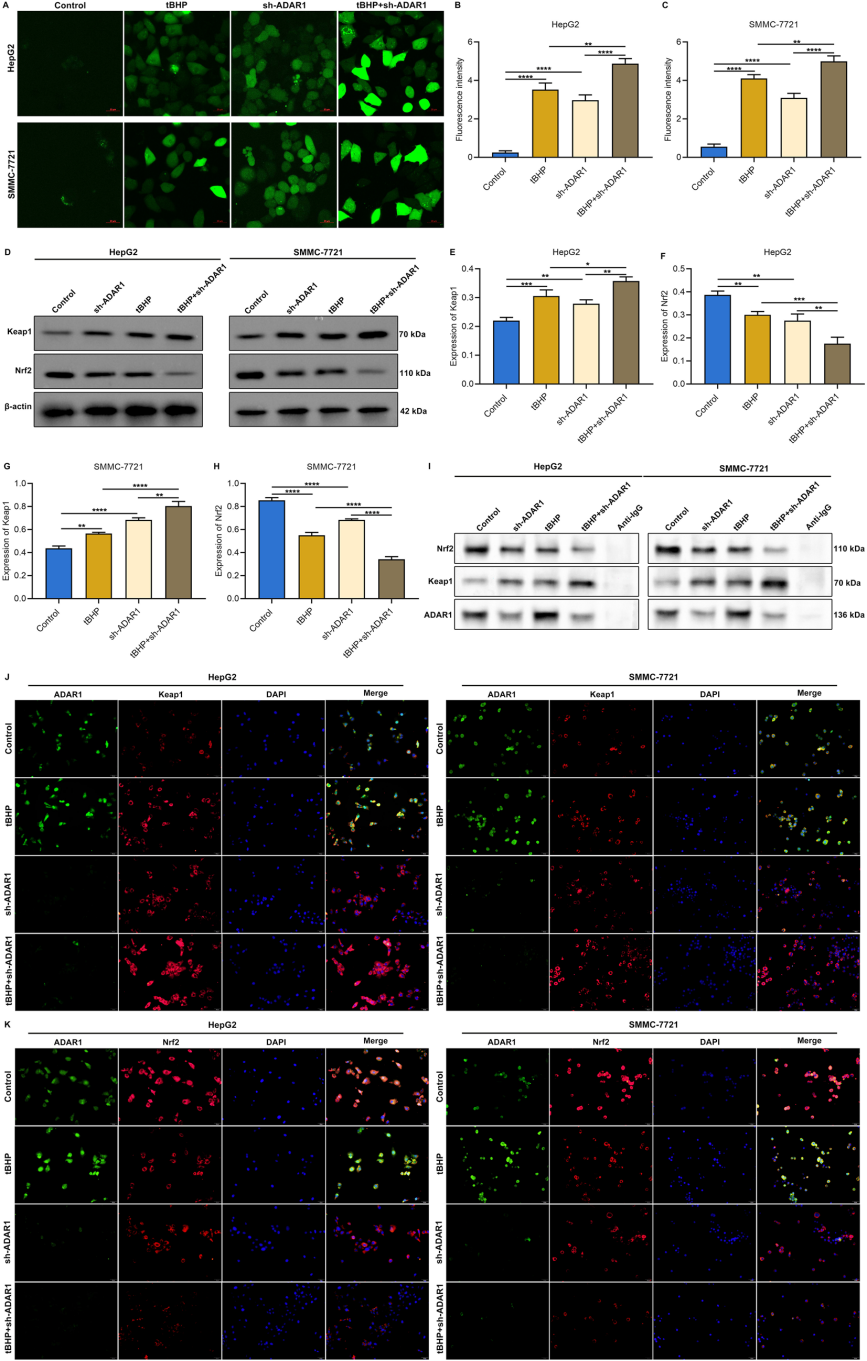
ADAR1 deficiency simultaneously heightens the effects of tBHP on intracellular ROS accumulation and regulation of Keap1/Nrf2 pathway in HCC cells. **A**–**C** DCFH-DA fluorescent probe for measuring intracellular ROS level of HepG2 and SMMC-7721 cells with tBHP administration and/or sh-ADAR1 transfection. Bar, 20 μm. **D**–**H** Immunoblot analysis of Keap1 or Nrf2 expression in HepG2 and SMMC-7721 cells with tBHP administration and/or sh-ADAR1 transfection. **I** Co-IP assay of Keap1 and Nrf2 in ADAR1 precipitates in HepG2 and SMMC-7721 cells with tBHP administration and/or sh-ADAR1 transfection. **J** Multiple immunofluorescence images of ADAR1 and Keap1 in HepG2 and SMMC-7721 cells with tBHP administration and/or sh-ADAR1 transfection. Bar, 10 μm. **K** Multiple immunofluorescence images of ADAR1 and Nrf2 in HepG2 and SMMC-7721 cells with tBHP administration and/or sh-ADAR1 transfection. Bar, 10 μm. *p < 0.05; **p < 0.01; ***p < 0.001; ****p < 0.0001

In accordance with analysis of DCFH-DA fluorescent probe, intracellular ROS level of HepG2 and SMMC-7721 cells was markedly heightened by tBHP administration (Fig. 6A–C). ADAR1 deficiency further reinforced intracellular ROS accumulation induced by tBHP administration. Moreover, we observed that tBHP treatment notably elevated Keap1 expression as well as lowered Nrf2 expression in HepG2 and SMMC-7721 cells, which were further aggravated by inhibition of ADAR1 (Fig. 6D–H). Co-IP assay proved that tBHP decreased the enrichment of Nrf2 and elevated the enrichment of Keap1 in HepG2 and SMMC-7721 cells, and the inducible phenomenon by oxidative stress was further aggravated by sh-ADAR1 in ADAR1 precipitates (Fig. 6I). As shown in immunofluorescence results, there was increased translocation of Keap1 as well as attenuated translocation of Nrf2 in HepG2 and SMMC-7721 cells with ADAR1 deficiency or tBHP treatment, and such phenomenon was further aggravated by the combination of both (Fig. 6J, K). Altogether, ADAR1 deficiency simultaneously heightened the effects of tBHP on intracellular ROS accumulation and regulation of Keap1/Nrf2 pathway in HCC cells.

### ADAR1 deficiency exacerbates the damage of tBHP on intracellular antioxidant system as well as destroys cellular morphology in HCC cells

**Fig. 7:**
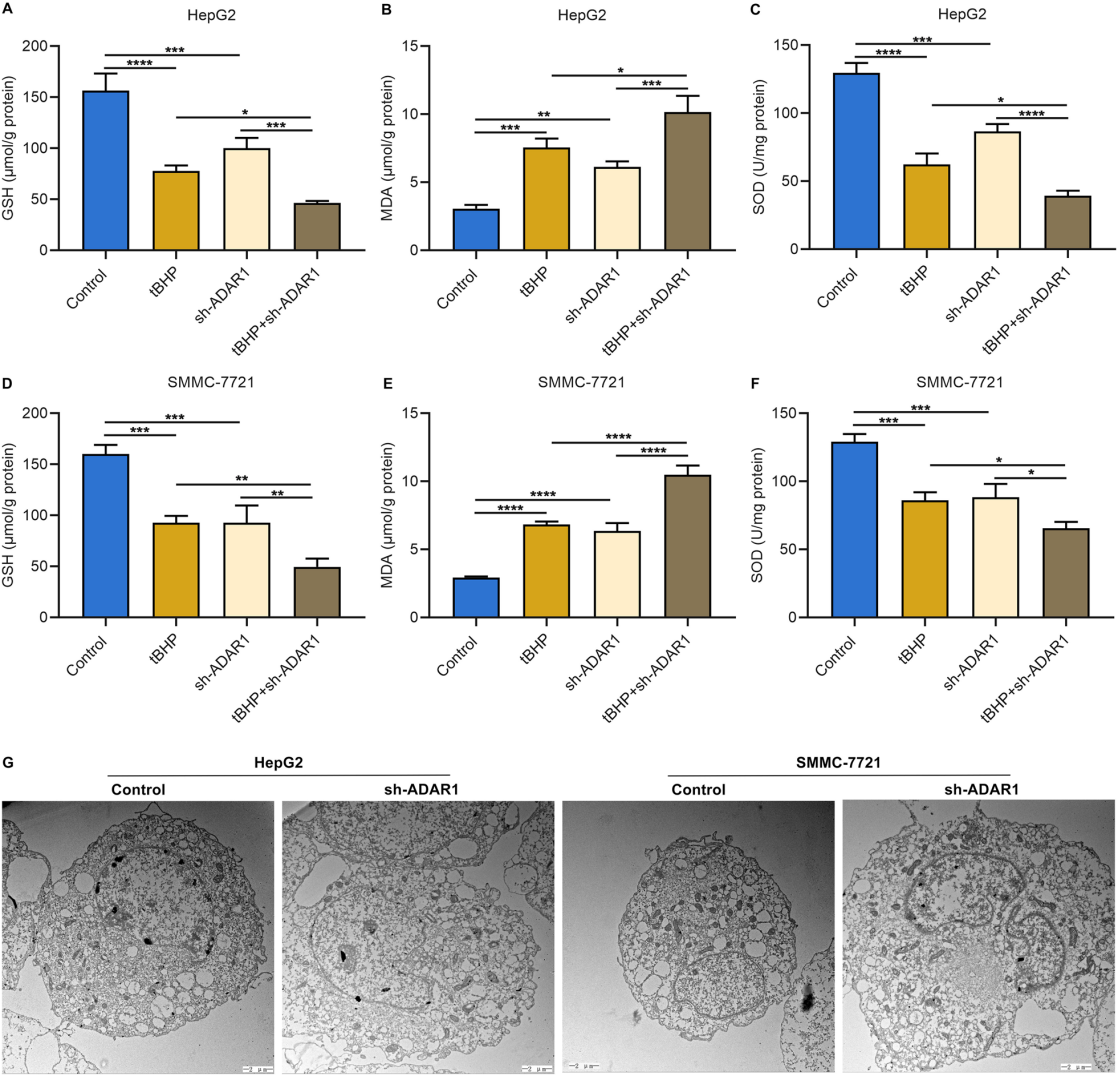
ADAR1 deficiency exacerbates the damage of tBHP on intracellular antioxidant system as well as destroys cellular morphology in HCC. **A**–**F** Measurement of intracellular content of GSH and MDA as well as activity of SOD in HepG2 and SMMC-7721 cells with tBHP administration and/or sh-ADAR1 transfection. **G** TEM images of changes in cellular morphology of HepG2 and SMMC-7721 cells with or without sh-ADAR1 transfection. Bar, 2 μm. *p < 0.05; **p < 0.01; ***p < 0.001; ****p < 0.0001

Both tBHP treatment and ADAR1 deficiency notably lowered intracellular GSH content and SOD activity as well as enhanced MDA activity in HepG2 and SMMC-7721 cells (Fig. 7A–F). ADAR1 deficiency simultaneously reinforced the effects of tBHP administration on intracellular content of GSH and MDA as well as the activity of SOD in HCC cells. Altogether, ADAR1 deficiency exacerbated the damage of tBHP on intracellular antioxidant system of HCC cells. In addition, we investigated the influence of ADAR1 deficiency on cellular morphology of HCC cells through TEM analysis. For normal HepG2 and SMMC-7721 cells, the cell morphology was complete, the nucleus was round and regular, the nuclear membrane was clear, the endoplasmic reticulum and mitochondria were normal (Fig. 7G). Oppositely, after ADAR1 deficiency, the cytoplasm and organelles were swollen, the chromatin was condensed, and mitochondria exhibited shrinkage, increased membrane density, reduced ridge, and rupture of the outer membrane (Fig. 7G).

### ADAR1 loss strengthens the inhibiting effect of sorafenib on HCC cell growth

**Fig. 8:**
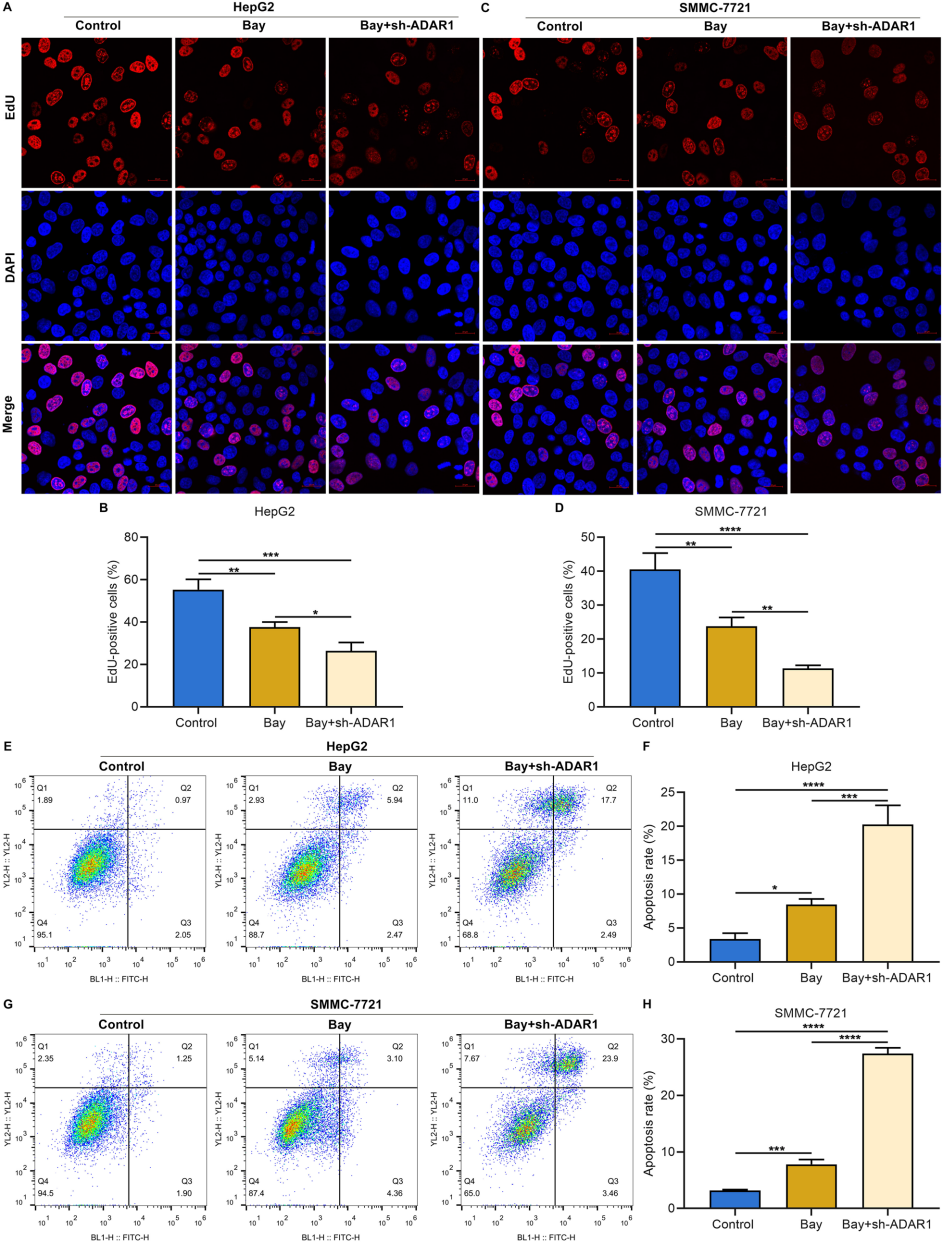
ADAR1 loss strengthens the inhibiting effect of sorafenib (Bay 43–9006) on HCC cell proliferation and the promoting effect on apoptosis. **A**–**D** EdU staining for proliferation of HepG2 and SMMC-7721 cells with Bay 43–9006 administration and/or sh-ADAR1 transfection. Bar, 20 μm. **E**–**H** Flow cytometry analysis of apoptotic level of HepG2 and SMMC-7721 cells with Bay 43–9006 administration and/or sh-ADAR1 transfection. *p < 0.05; **p < 0.01; ***p < 0.001; ****p < 0.0001

Sorafenib (Bay 43–9006), an oral multikinase inhibitor, has been put into the standard of care for patients with advanced HCC [37]. As expected, BAY notably mitigated proliferative capacity of HepG2 and SMMC-7721 cells (Fig. 8A–D). Combination of BAY and ADAR1 deficiency simultaneously attenuated HCC cell proliferation. Additionally, apoptotic level of HepG2 and SMMC-7721 cells was induced by BAY administration (Fig. 8E–H). ADAR1 loss simultaneously aggravated BAY-triggered HCC cell apoptosis.

### ADAR1 loss simultaneously strengthens the effects of sorafenib on intracellular ROS accumulation and regulation of Keap1/Nrf2 pathway in HCC cells

**Fig. 9:**
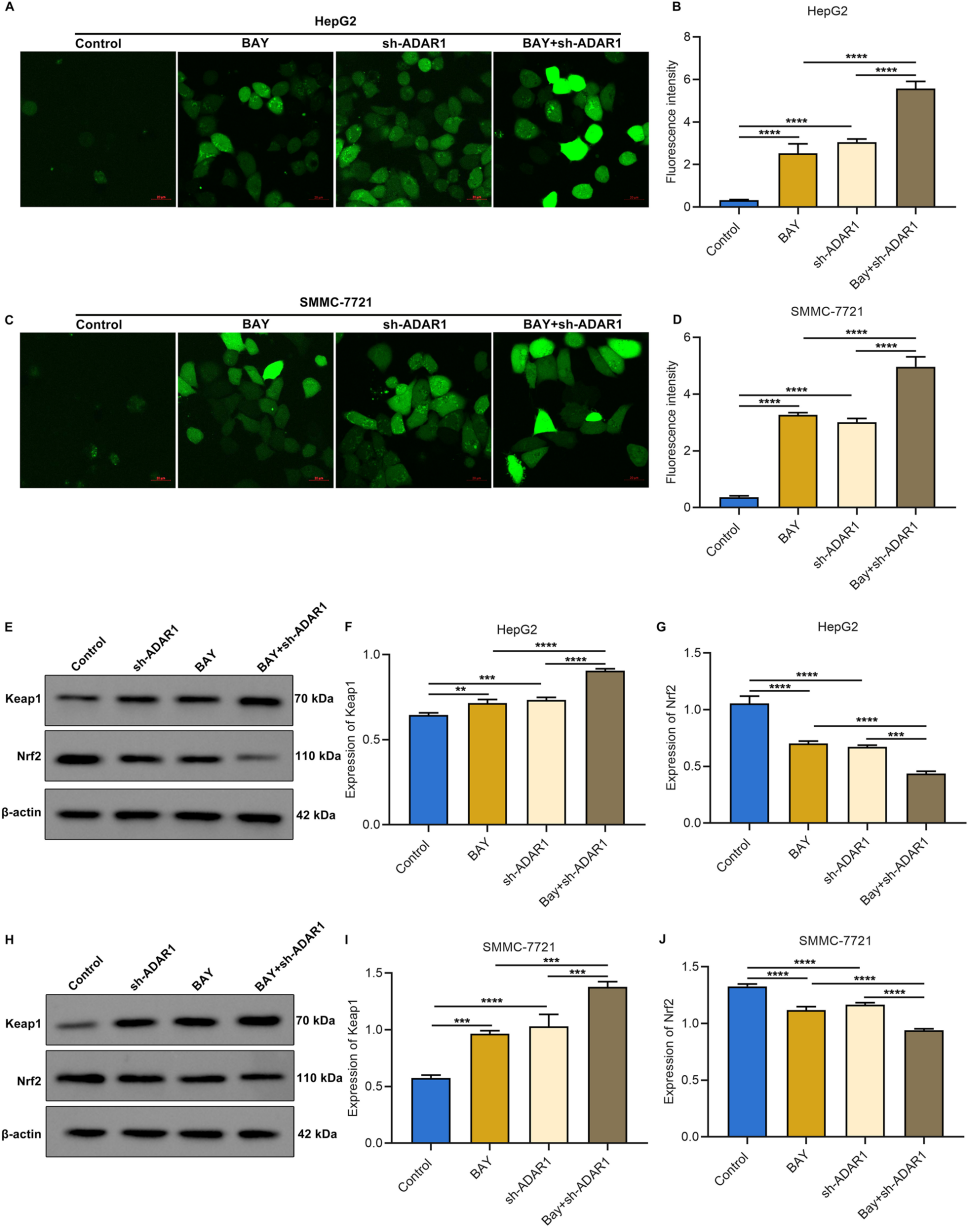
ADAR1 loss simultaneously strengthens the effects of sorafenib (Bay 43–9006) on intracellular ROS accumulation and regulation of Keap1/Nrf2 pathway in HCC cells. **A**–**D** DCFH-DA fluorescent probe for detecting intracellular ROS level of HepG2 and SMMC-7721 cells with Bay 43–9006 exposure and/or sh-ADAR1 transfection. Bar, 20 μm. **E**–**J** Immunoblot analysis of Keap1 or Nrf2 expression in HepG2 and SMMC-7721 cells with Bay 43–9006 exposure and/or sh-ADAR1 transfection. **p < 0.01; ***p < 0.001; ****p < 0.0001

**Fig. 10:**
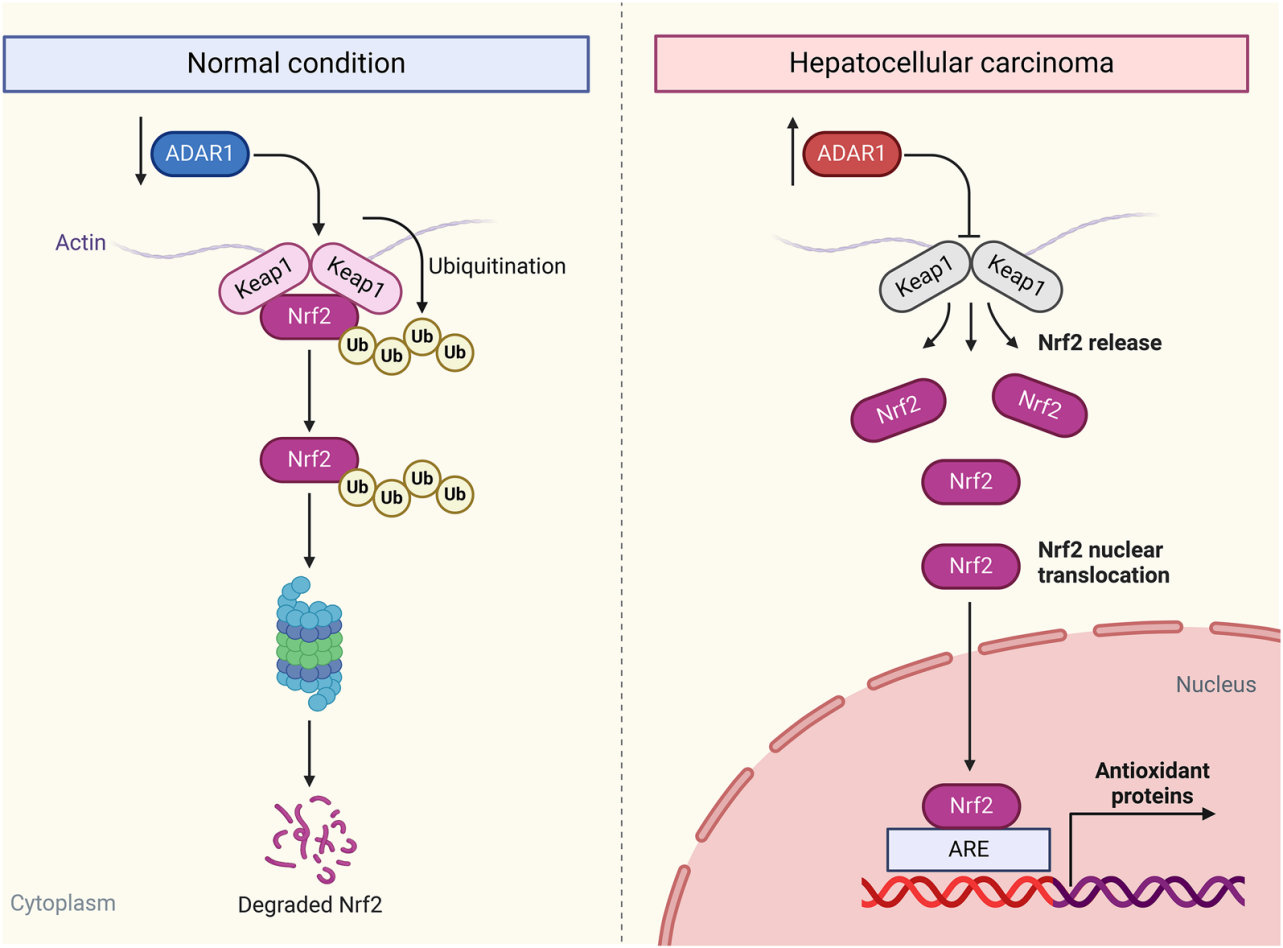
Schematic illustration depicting the role and molecular mechanisms of ADAR1 in HCC. Suppressing ADAR1 may sensitize HCC cells to oxidative stress via modulating Keap1/Nrf2 pathway

Evidence suggests that ROS accumulation is a critical mechanism of sorafenib-induced cell deaths in HCC [38]. Consistently, our data demonstrated that BAY observably elevated intracellular ROS accumulation of HepG2 and SMMC-7721 cells. Simultaneous ADAR1 loss further exacerbated intracellular ROS accumulation in BAY-exposed HCC cells (Fig. 9A–D). Sorafenib may induce Nrf2 degradation and facilitate subsequent Nrf2 nuclear translocation through activating Keap1 in HCC [39]. As expected, BAY exposure markedly elevated Keap1 expression as well as lowered Nrf2 expression in HepG2 and SMMC-7721 cells (Fig. 9E–J). ADAR1 loss in combination with BAY remarkably improved Keap1 expression and attenuated Nrf2 expression in HCC cells. Taken together, our study demonstrated that suppression of ADAR1 may sensitize HCC cells to oxidative stress through regulating Keap1/Nrf2 pathway (Fig. 10).

## Discussion

The accumulation of genetic and epigenetic alterations contributes to the occurrence and development of HCC [40]. RNA editing is a broad process of co- or post-transcriptional modification event introducing alterations in genome-encoded RNA sequences [41]. A to I in dsRNA, catalyzed by ADAR family enzymes (ADAR1-3), represents the most frequent RNA editing type in humans. Dysregulated A-to-I RNA editing is found in HCC [42]. Oxidative stress is a major cause in HCC development. The present study offered evidence that ADAR1 was essential for survival and oxidative stress of HCC cells, and targeting ADAR1 may sensitize HCC cells to oxidative stress via mediating Keap1/Nrf2 pathway.

Our pan-cancer analysis unveiled the up-regulation of ADAR1 in most cancer types, especially HCC. Consistent with previous research [43], ADAR1 was frequently overexpressed in HCC. Additionally, higher ADAR1 expression was observed in metastatic HCC. Experimental evidence has demonstrated that ADAR1 loss may suppress peritoneal metastasis of gastric cancer via Wnt/β-catenin pathway [26]. Activation of AZIN1 RNA editing by ADAR1 facilitates invasive capacity of cancer-associated fibroblasts in colorectal carcinoma [44]. Numerous prognostic markers (such as SGOL2 [45] and circRanGAP1 [46]) for HCC have been discovered, but none of them have been applied in clinical practice. Up-regulated ADAR1 is correlated to worse clinical outcome for HCC cases [22]. Larger prospective cohorts are needed to assess whether ADAR1 may act as a prognostic biomarker of HCC. In HCC cells, ADAR1 deficiency mitigated proliferation and tumor growth and enhanced apoptosis, indicating that ADAR1 was essential for HCC progression. Except for HCC, ADAR1 plays a pro-tumorigenic role in glioblastoma through a RNA editing-independent mechanism [47], and ADAR1-mediated RNA editing correlates ganglioside catabolism to stem cell maintenance for glioblastoma [48]. ADAR1 enables to mask the cancer immunotherapeutic promise of ZBP1-triggered necroptotic cell deaths [49]. Additionally, ADAR1 loss strengthens the sensitivity of non-small cell lung cancer cells to anlotinib through modulating CX3CR1-fractalkine expression [50].

The imbalance between ROS production and elimination results in the moderate oxidative stress frequently found in HCC [51]. Tumor cells are distinguished from normal cells by the capacity to generate increased ROS level and enhanced dependence on antioxidant defense system [51]. Thus, targeted modulation of antioxidant ability of tumor cells possesses a potent therapeutic impact [52]. In HCC cells, intracellular ROS accumulation was strengthened by ADAR1 deficiency. Nrf2 is a master regulator of a variety of antioxidant enzymes, and its constitutive stabilization and activation results in worse clinical outcome for HCC [53]. Following a physiological condition, Nrf2 activity is restricted through binding to Keap1 in the cytoplasm, thus limiting its translocation to the nucleus. As such, low constitutive level of Nrf2 is essential for maintaining a basal antioxidant level, and Keap1-Nrf2-ARE signaling protects the cells against oxidative stress. When cellular redox homeostasis is recovered, Keap1 is translocated into the nucleus, thus releasing Nrf2 from the AREs [54]. Under an oxidative stress condition, oxidative stress agonist tBHP and sorafenib (Bay 43–9006) both elevated Keap1 expression, resulting in inactivating Nrf2 as well as its downstream targets. ADAR1 deficiency disrupted redox homoeostasis and sensitized HCC cells to oxidative stress triggered by tBHP and sorafenib via activating Keap1 and inactivating Nrf2. Acquired or intrinsic resistance of HCC cells to apoptosis is capable of limiting the induction of apoptotic cell deaths by sorafenib. Thus, targeting ADAR1 might be an underlying strategy to overcome resistance of targeted therapy as well as boost HCC therapy.

## Conclusions

In the current study, we firstly determined ADAR1 as a crucial mediator of sensitizing HCC cells to oxidative stress. ADAR1 deficiency resulted in tumor survival suppression, apoptosis, and oxidative stress (with enhanced intracellular ROS accumulation, reduced intracellular GSH content and SOD activity and increased MDA content) of HCC cells by mediating Keap1-Nrf2 signaling. As such, ADAR1 represents a potent target to sensitize HCC cells to oxidative stress triggered by targeted therapy. Hence, inhibitors of ADAR1 warrant further clinical assessment.

## Data Availability

The datasets analyzed during the current study are available from the corresponding author on reasonable request.

## Abbreviations

HCC: Hepatocellular carcinoma
ROS: Reactive oxygen species
AREs: Antioxidant response elements
ADAR: Adenosine deaminase acting on RNA
dsRNA: Double-stranded RNA
FBS: Fetal bovine serum
BCA: Bicinchoninic acid
shRNA: Small hairpin RNA
tBHP: Tert-butyl Hydroperoxide
CCK-8: Cell counting Kit-8
EdU: 5-Ethynyl-2′-deoxyuridine
GSH: Glutathione
MDA: Malondialdehyde
SOD: Superoxide dismutase
TCGA: The Cancer Genome Atlas
ANOVA: One-way analysis of variance
